# Exploring the Therapeutic Ability of Fenugreek against Type 2 Diabetes and Breast Cancer Employing Molecular Docking and Molecular Dynamics Simulations

**DOI:** 10.1155/2018/1943203

**Published:** 2018-07-11

**Authors:** Shailima Rampogu, Saravanan Parameswaran, Mary Rampogu Lemuel, Keun Woo Lee

**Affiliations:** ^1^Division of Life Science, Division of Applied Life Science (BK21 Plus), Plant Molecular Biology and Biotechnology Research Center (PMBBRC), Research Institute of Natural Science (RINS), Gyeongsang National University (GNU), 501 Jinju-daero, Jinju 52828, Republic of Korea; ^2^West Thames College, London, UK

## Abstract

Fenugreek (Trigonella foenum-graecum) is used as a spice throughout the world. It is known for its medicinal properties such as antidiabetic, anticarcinogenic, and immunological activities. The present study shows the properties and the nutritional quality of fenugreek seed extract and focuses on screening of active compounds in drug designing for type 2 diabetes and breast cancer. Quantitative analysis was used to calculate the percentages of protein, carbohydrates moisture, fatty acid, galactomannan, oil, and amino acid. Phytochemical analysis revealed the presence of flavonoids, terpenoids, phenols, proteins, saponins, and tannins in fenugreek seed extracts. Molecular docking and molecular dynamics simulation-based computational drug discovery methods were employed to address the role of fenugreek seed constituents against type 2 diabetes and breast cancer. The computational results reveal that the compound galactomannan can be ascribed as potential drug candidate against breast cancer and type 2 diabetes rendered by higher molecular dock scores, stable molecular dynamics (MD) simulations results, and lower binding energy calculations.

## 1. Introduction

The legume fenugreek* (Trigonella foenum-graecum) *is a short annual plant from the Fabaceae family [1, 2]. The name Trigonella foenum-graecum is a Latin-Greek name as it bears a typical triangular shaped flowers and is employed as a common fodder for animals in Greece [1]. It is found in various parts of the globe and is often used as spice, condiment, and medication [3, 4]. Largely, fenugreek leaves and seeds have been used as spices in different parts of the globe. In Africa, fenugreek is used as supplement during bread preparation and the seed components of fenugreek are known to enhance the nutritional quality of the bread. In India, the leaves and seeds are utilized as favouring and seasoning agents [1]. In China, it is used as cure edema, while the ancient Egyptians employed fenugreek to incense the mummies [1, 5, 6]. Additionally, fenugreek is used as a medicine to treat several diseases besides being used as antioxidant [7], against inflammation [8, 9], as anticancer [10], as hepatoprotective agent [11, 12], as antibacterial [13–15], and as antifungal [16]. Additionally, fenugreek is also used as off-season fodder and animal food supplement [17].

Fenugreek seeds are widely studied part of the plant. The powdered fenugreek is used as condiment and the seed endosperm serves to secure fenugreek gum [1]. The seeds have a strong aroma with bitter taste [18]. The major chemical constituents found in fenugreek seed are galactomannan (fibre), diosgenin (sapogenin), trigonelline (alkaloid), and 4-hydroxyisoleucine that have the antidiabetic properties and are also employed to treat breast cancer [19].

Diabetes mellitus is a common and chronic disease concern globally associated with a ten-year shorter life expectancy [20]. According to WHO, type 2 diabetes occurs because either body does not produce enough insulin or body resists the effects of insulin [21, 22]. Type 2 diabetes is dominant in developing countries and accounts to around 85%–90% worldwide [20, 21]. Fenugreek is another promising antidiabetic drug [23]. It was also confirmed that consuming fenugreek as a dietary supplement in the prediabetic patients could efficiently reduce the outbreak of type 2 diabetes [24]. Additionally, it was further reported that the socked fenugreek seeds can act as adjuvant in mitigating the type 2 diabetes and also in noninsulin dependent diabetes [25, 26] and serum lipids in type I diabetes [27]. Additionally, it is well evidenced that the fenugreek seeds are antidiabetic in nature [24, 28, 29].

Fenugreek also possesses anticancer properties and chemical constituents of fenugreek are known to induce apoptosis [30, 31]. Furthermore, it induces dose-dependent effect on human breast cancer cell line [32]. Breast cancer is the most common cause of death in female worldwide [33, 34]. The discovery of BRCA1 and BRCA2 genes helped to understand that hereditary factor is the main cause of most cancers [35]. Chloroform seed extract studies by Khoja* et al.* proved the effective killing of MCF-7 human immortalized breast cells [30]. Amin* et al.* (2005) studies suggest that fenugreek seed chemical constituents have preventive effect against breast cancer which inhibit MDA 231-induced mammary hyperplasia [36]. However, it is not yet delineated on the most effective compound that can act on both the morbidities. Therefore, in the current investigation, we employed the computational technique such as molecular docking and molecular dynamics simulations to identify candidate compound as compared with the reference compounds.

Molecular docking is one of the widely adapted methods to predict the binding affinities between the ligand and the target protein and further the lead optimization [37]. Additionally, the molecular docking imparts knowledge on the interactions at the atomic level [37] and predicts the ideal binding mode [33, 38]. Molecular docking mechanism generally evaluates the binding conformations, its orientation, and the accommodation of the small molecule at the active site of the proteins binding site and are read as scores [39]. The molecular dynamics simulation imparts knowledge on the nature of the small molecules at the proteins binding pocket thereby affirming the appropriate binding modes [38]. The identified Hits that have demonstrated a higher dock score than the reference compounds or the known drugs, exhibiting the interactions with the key residues complemented by stable molecular dynamics simulation results, are considered the most promising candidate compounds.

In the current investigation, the quantitative analysis of fenugreek seeds was conducted to gain information on the components and further the computational analysis was performed to discover the potential compound against breast cancer and type 2 diabetes. The* in silico *results have illuminated galactomannan as the prospective compound against both diseases.

## 2. Materials and Methods

Fenugreek seeds were used as a sample to test the medicinal properties. Fenugreek seeds were sourced from a local market (Hyderabad, India) and were of high quality grade. They were shade dried, cleaned, and finely powdered and used for chemical analysis.

### 2.1. Biochemical Analysis

The biochemical studies were carried out to identify the protein content, total soluble carbohydrates, oil content and fatty acid values, free amino acids, and soluble fibres from the collected seed samples.

#### 2.1.1. Estimation of Total Protein

Percentage of proteinaceous nitrogen and proteins was estimated by the Micro-kjeldahl method [40]. Proteinaceous nitrogen was calculated by the following formula.

(1) $$\begin{array}{c} \%\;\;of\;\;Nitrogen=\left(\;T-B\;\right)\times N\times 10\times \frac{1.4}{28}\times S \end{array}$$

  T is titration reading of the sample,
  B is blank reading of the sample,
  S is the amount of sample taken in grams,
  N is normality of hydrochloric acid (N/28).

To calculate the percentage of protein, the nitrogen value was multiplied by the factor 6.25.

#### 2.1.2. Estimation of Total Carbohydrate

Total carbohydrate content of the seed samples was estimated by the procedure suggested by Loewis (1952) [41]. Anthrone reagent was used and the developed colour was read at 620nm in a colorimeter against blank.

#### 2.1.3. Estimation of Oil Content

Total oil content of the said spices was estimated as suggested by Meara (1955) [42].

Percentage of oil was calculated by following formula:

(2) $$\begin{array}{c} \%\;\;of\;\;oil=\frac{Wo}{Ws}\times 100 \end{array}$$

  Wo is the weight of oil extracted,
  Ws is the weight of seed taken.

#### 2.1.4. Estimation of Fatty Acid Value

Method used to estimate the fatty acid value is suggested by Meara (1955) [42].

Fatty acid value was calculated using the formula

(3) $$\begin{array}{c} Fatty\;\;acid\;\;value=U\times \frac{56.1}{W} \end{array}$$

  U is the volume of titration of 0.1 n KOH,
  W is the grams of oil taken.

#### 2.1.5. Isolation of Amino Acids

Column chromatography was used to isolate free amino acids from fenugreek seeds [43].

To find the concentration of 4-hydroxyisoleucine, first the total amino acid content was determined by using spectrophotometric method. Then the relative concentration of 4-hydroxyisoleucine in the mixture of amino acid was determined by high performance thin layer chromatography (HPTLC).

#### 2.1.6. Isolation of Galactomannans

Extraction and isolation of the water-soluble polysaccharides (galactomannans) from endosperm of fenugreek seeds were done using the procedure of Kooiman (1971) [44].

#### 2.1.7. Estimation of Moisture Percentage

Moisture content of seeds was estimated by “Dry air oven” method association of official analytical chemists (AOAC) (anonymous, 1947)[45] and the percentage was calculated from the following formula:

(4) %  moisture=fresh  weight  of  the  seed−dry  wt.  of  the  seeddry  wt.  of  the  seed×100

### 2.2. Molecular Docking, Simulations, and Free Energy Calculations

To further assess the suitability of the compounds as antidiabetic and potential breast cancer agents, the investigation proceeds employing the computational methods such as molecular docking recruiting CDOCKER available on Discovery Studio (DS) v4.5, molecular dynamics (MD) simulations conducted using GROningen MAchine for Chemical Simulations (Gromacs) v5.0, which was followed by MM/PBSA calculations.

#### 2.2.1. Molecular Docking

For the execution of the docking protocol, the proteins for both the diseases were imported from protein data bank (PDB) of high resolution. The protein with the PDB id 3EQM (2.9Å) was chosen for breast cancer and 1GFY (2.1Å) was elected for type 2 diabetes, respectively. These proteins were prepared on DS by initiating the* clean protein* module embedded with the DS and subsequently heteroatoms together with the water molecules were dislodged and the addition of hydrogens was performed adapting the CHARMm force field accessible on the DS. The active sites were selected in accordance with the co-crystal geometry, thereby, considering the residues around 10 Å radius [46, 47].

Phytochemicals along with the type 2 diabetic and breast cancer drugs, canagliflozin [48] and anastrozole [49], were used to comparatively evaluate the effect of the prospective drug molecules on the diseases labelling the latter as reference drug. These compounds were imported onto the DS to obtain their 3D structures and were subsequently minimized. The prepared proteins and the ligands were subjected to molecular docking studies employing the CDOCKER protocol.

CDOCKER available on the DS happens to be the most reliable method as it employs the CHARMm-based dynamics methods [50]. Subsequently, 30 conformations were allowed to be generated for each ligand, while the other parameters were set at default. The results were evaluated based upon the higher –CDOCKER interaction energy and higher –CDOCKER energy that significantly correspond to the favourable binding. The most appropriate binding mode was judged by the maximum clusters formed and was therefore subjected to MD simulations to understand its dynamic behaviour.

#### 2.2.2. MD Simulations

Molecular dynamics (MD) simulations were performed for the favourable systems obtained after docking using GROMACS 5.0 with CHARMm27 force field. Ligand topologies were generated adapting the SwissParam [51]. All the parameters were attributed as described earlier [52–56]. Dodecahedron water box was generated and the systems were solvated comprising three-site transferrable intermolecular potential (TIP3P) water model, to which the counter ions were added. The system was energy minimized with steepest descent algorithm with 10000 steps which was then subjected to equilibration using constant number N, volume V, and temperature T (NVT) [57] and constant number N, pressure P, and temperature T (NPT) [58]. During this process, the protein backbone was restrained and the periodic boundary conditions were fostered to avoid bad effects. Thereafter, the MD run was conducted for 10 ns, saving the data for every one picosecond (ps). Visual molecular dynamics (VMD)[59] and DS were utilized to analyse the MD results.

#### 2.2.3. Binding Free Energy Calculations

Molecular Mechanics/Poisson Boltzmann Surface Area (MM/PBSA) was recruited to compute the binding free energy calculations [60, 61]. 10 snapshots were evenly extracted from the MD trajectories of the protein ligand complex. A variety of energetic values were calculated using

(5) ΔGbinding=Gcomplex−Gprotein+GligandGX=EMM+GsolvationEMM=Ebonded+Enon-bonded=Ebonded+Evdw+EelecGsolvation=Gpolar+Gnon-polarGnon_polar=γSASA+b

## 3. Results

### 3.1. Biochemical Analysis

The total seed percentage revealed that galactomannan and 4-hydroxyisoleucine were present in 26.4 and 13 percentages, respectively, as in Table 1.

Further phytochemical screening of acetone seed extract of fenugreek was carried out to test the presence of tannins, phenols, terpenoids, flavonoids, saponins, and alkaloids [62] and are tabulated in Table 2.

***Test for flavonoids***: 1 ml of extract in a test tube and 5ml of diluted ammonium solution were added followed by few drops of concentrated sulphuric acid. Formation of yellow colour indicated the presence of flavonoids [62].

***Test for tannins:*** Formation of reddish-brown colour indicated the presence of tannins (ferric chloride test) when 1% ferric chloride solution was added to 1 ml of extract of fenugreek seeds [62].

***Test for terpenoids:*** To find out the presence of terpenoids, Salkowski test was conducted. 1 ml of extract was taken and dissolved in chloroform and then a few drops of concentrated sulphuric acid were added to it. On the inner face, a reddish-brown colour was formed that indicated the presence of terpenoids [62].

***Test for alkaloids*** *:* Dragendorffa's test results indicated the presence of alkaloids by giving orange-red precipitate, when 1 ml of Dragendroffa's reagent was added (potassium bismuth iodide solution) to 1 ml of extract [62].

***Test for saponins*** *:* Frothing test was conducted to test for saponins in the seed extract. 1ml of extract was vigorously shaken with distilled water and was allowed to stand for 10 min. Stable froth indicated the presence of saponins [62].

### 3.2. Molecular Docking, Simulations, and Free Energy Calculations

#### 3.2.1. Molecular Docking Studies

Molecular docking was executed independently for diabetes and breast cancer. The ligands along with their respective proteins were docked to assess their binding affinities. It was interesting to note that 4-hydroxyisoleucine has generated a relatively lower dock score while galactomannan produced higher dock score as compared to their respective reference compounds, as in Table 3. Therefore, 4-hydroxyisoleucine was refrained from further calculations and the other systems were proceeded forward.

#### 3.2.2. Molecular Dynamics Simulations

**Figure 1:**
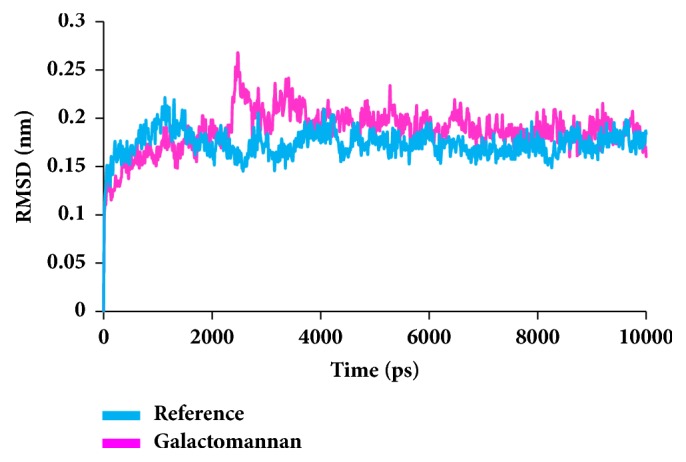
RMSD plots for backbone atoms.

**Figure 2:**
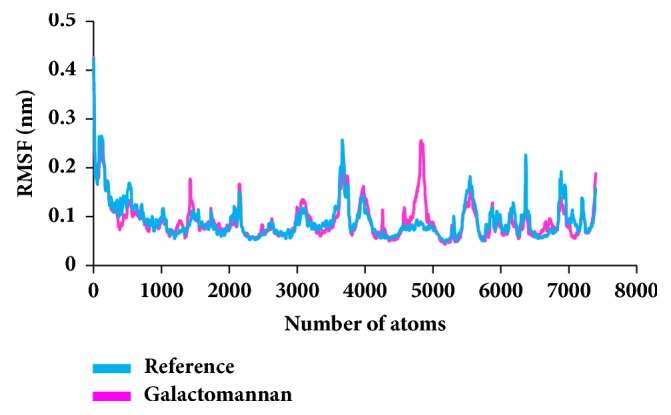
RMSF profiles for backbone atoms.

**Figure 3:**
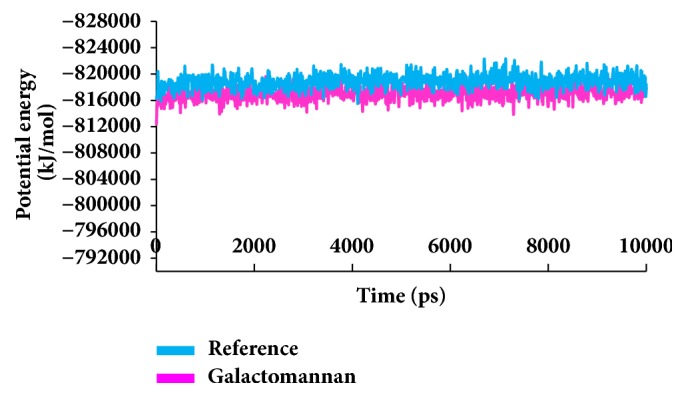
Potential energy graphs of the systems.

**Figure 4:**
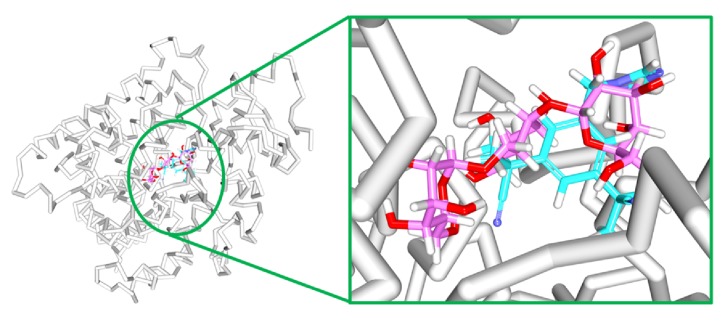
Binding mode assessment of the reference (cyan) and galactomannan (pink). Superimposition of the representative structures (left) and zoomed (right).

**Figure 5:**
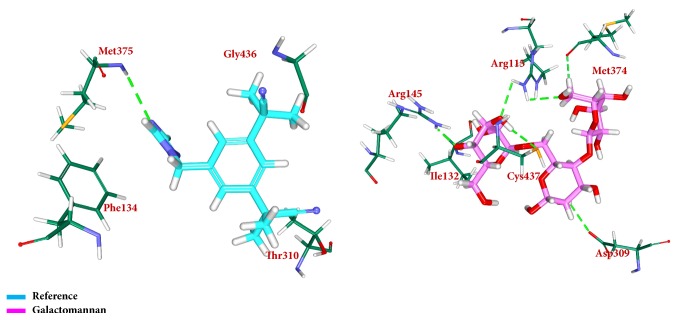
Depiction of hydrogen bond interactions and binding conformations. Only polar atoms are displayed for clarity.

**Figure 6:**
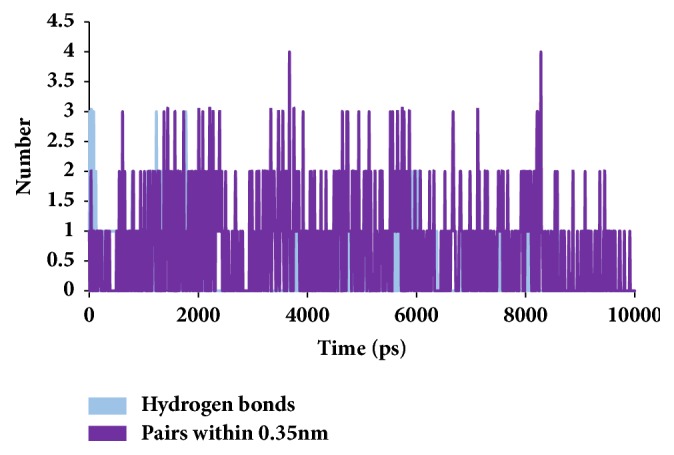
Graphical depiction of number of intermolecular hydrogen bond interactions between the protein and the reference compound.

**Figure 7:**
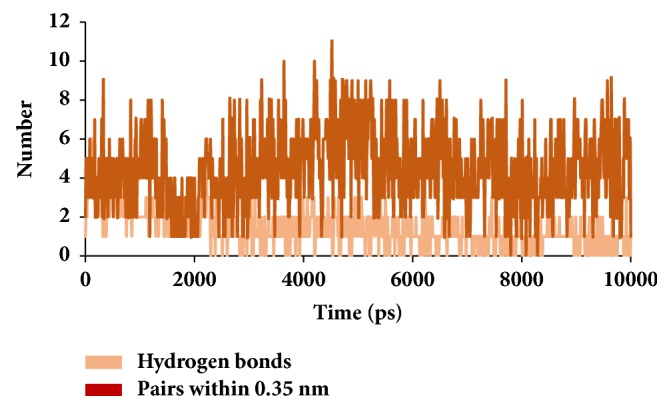
Graphical depiction of number of intermolecular hydrogen bond interactions between the protein and the candidate compound.

To secure the results obtained from the docking, the MD simulations were performed to establish the most reliable ligand-receptor complex and additionally to understand their behaviour at proteins active site [52, 53]. The MD for 10 ns was initiated and the behaviour of each system was monitored. Accordingly, root mean square deviation (RMSD), root mean square fluctuation (RMSF), and potential energies were calculated for each system. The RMSD for the breast cancer systems were observed to be stable after 4000 ps with no significant variation, thereafter, implying that the system is well converged, as in Figure 1. Moreover, the RMSD values were demonstrated to be less than 0.25 nm. Similar results were noted with RMSF values as well, as in Figure 2. The potential energy further states that there were no abnormal behaviours of the systems which were stable throughout the simulations, as in Figure 3. The last 5ns trajectories were retrieved to study the binding mode analysis. Upon superimposition, it was conceived that the binding mode pattern of the reference and the galactomannan were similar, as in Figure 4. The interactions of the ligand with the protein were evaluated with the key residues located at the active site. The reference compound anastrozole was seen to form a hydrogen bond with the NH atom of Met374 residue, joined by N5 atom with a bond length of 2.9 Å. Phe134 was found to form the *π* – *π* with the ligand molecule. Galactomannan was found to interact with the protein by forming 7 hydrogen bonds. The O13 atom of the ligand has interacted with the HH22 atom of Arg115 with a bond length of 2.8 Å. The HH21 atom of Arg115 has interacted with O15 atom of the ligand with a bond length of 2.5 Å. The O atom of Ile132 has joined with H62 of the ligand displaying a bond length of 2.6 Å. Another hydrogen bond was observed between the HH11 atom of Arg145 and the O14 atom of the ligand with a length of 2.0 Å. The OD2 atom of the residue Asp309 has interacted with the H57 of the ligand with a bond distance of 2.8 Å. The O atom of the key residue Met374 has interacted with the H53 atom of the ligand with a bond length of 2.5 Å. The SG atom of the Cys437 residue has interacted with the H63 atom of the ligand with a distance of 2.5 Å. The details of the interaction are represented in Figure 5 and Table 4. Furthermore, the intermolecular hydrogen bond interactions were recorded during the simulations to elucidate deposition of the ligand within the active site. It was observed that the reference molecule displayed an average of 0.3 hydrogen bonds, while those within 0.35 nm were observed to be 0.7, as in Figure 6, while the candidate molecule demonstrated an average of 1.3 hydrogen bonds and the bonds within 0.35 nm were 4.4, as in Figure 7.

**Figure 8:**
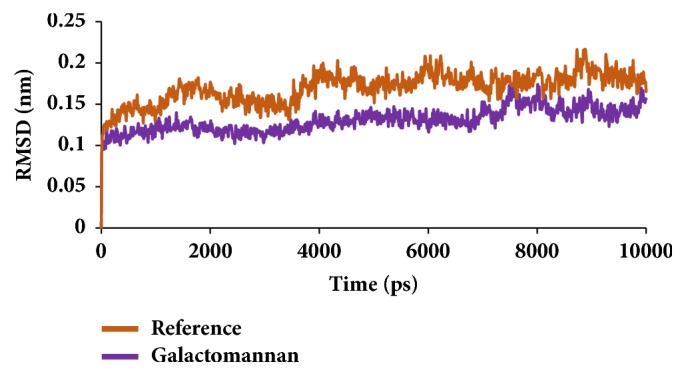
RMSD plots for backbone atoms.

**Figure 9:**
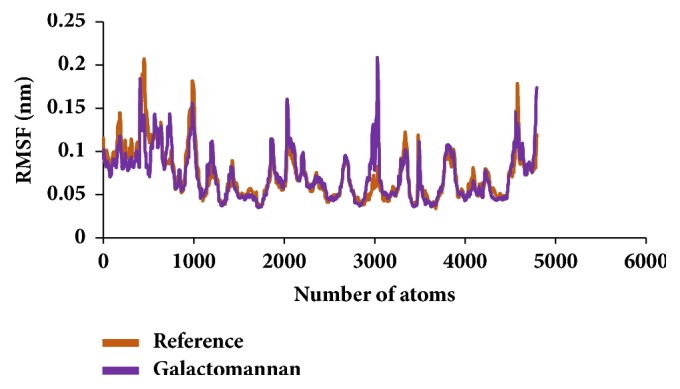
RMSF profiles for backbone atoms.

**Figure 10:**
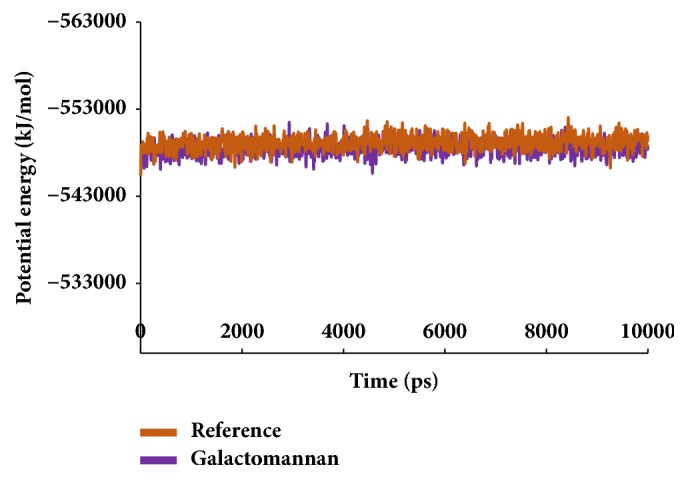
Potential energy graphs of the systems.

**Figure 11:**
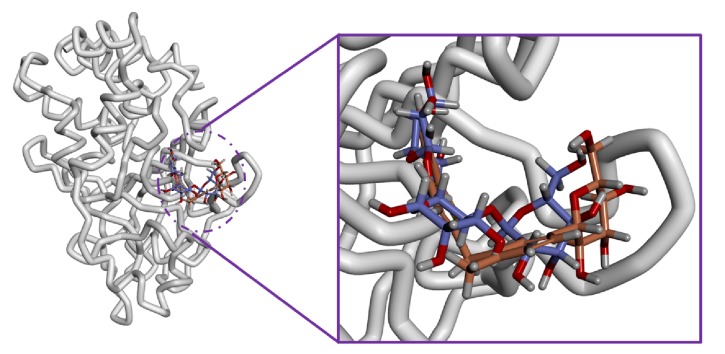
Binding mode assessment of the reference (purple) and galactomannan (orange). Superimposition of the representative structures (left) and zoomed (right).

**Figure 12:**
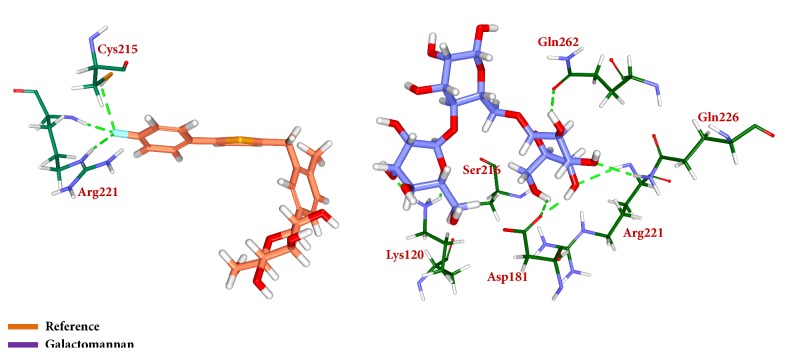
Depiction of hydrogen bond interactions and binding conformations. Only polar atoms are displayed for clarity.

**Figure 13:**
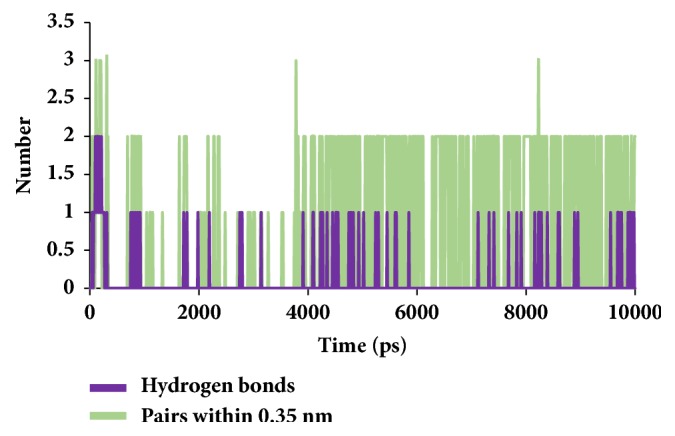
Graphical depiction of number of intermolecular hydrogen bond interactions between the protein and the reference compound.

**Figure 14:**
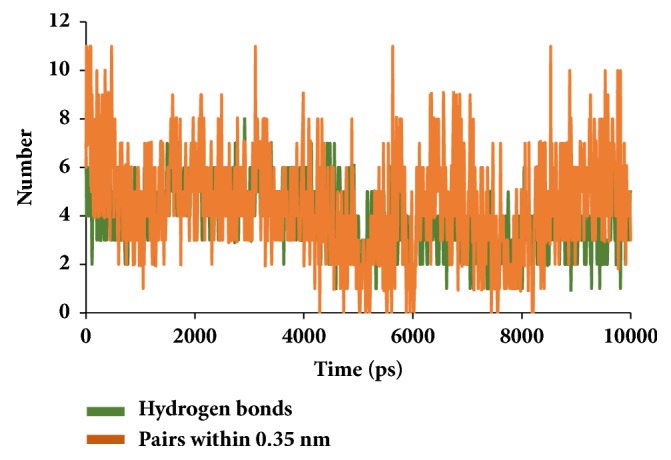
Graphical depiction of number of intermolecular hydrogen bond interactions between the protein and the candidate compound.

Similar types of calculations were determined for the type 2 diabetes disease target and its respective ligands. The RMSD was recorded to be stable after 7000 ps for both the reference and galactomannan. Further, it was noted that the RMSD of the reference was established to be within 0.2 nm while the drug-like molecule demonstrated a RMSD within 0.15 nm, as in Figure 8. However, no major fluctuations were noticed during the simulations referring to the stability of the systems. The same results were depicted through the RMSF, as in Figure 9, and the potential energy calculations, as in Figure 10. Therefore, to examine the binding mode of the ligand molecules, the last 5 ns trajectories were extracted and were superimposed. The results represented a similar binding mode between the reference and the galactomannan, as in Figure 11. Furthermore, intermolecular interactions were inspected with the key residues residing at the active site. It revealed that the reference molecule has formed three hydrogen bonds with the active site residues. The F2 of the ligand has interacted with the HG atom of Cys215 with bond length of 2.6 Å. The other two hydrogen bonds are formed with HN and HE atoms of Arg221 and 2.1 Å each. Tyr46 and Phe182 have been involved with the *π* – *π* interactions. On the contrary, Galactomannan on the other hand generated eight hydrogen bonds, two hydrogen bonds with Lys120 and Asp181 amino acid residues and one hydrogen bond with Arg221, Ser216, Gln262, and Gln266, respectively. The details of the interactions are tabulated in Figure 12 and Table 5. Furthermore, the intermolecular hydrogen bonds were evaluated throughout the simulations. The average hydrogen bonds were computed to be 0.09 and those within 0.35 nm were found to be 0.7, as in Figure 13. The prospective drug molecule however has represented average hydrogen bonds of 3.9 while the bonds within 0.35 nm were enumerated to be 4.4 projecting the superiority of galactomannan, as in Figure 14.

### 3.3. Binding Free Energy Analysis

**Figure 15:**
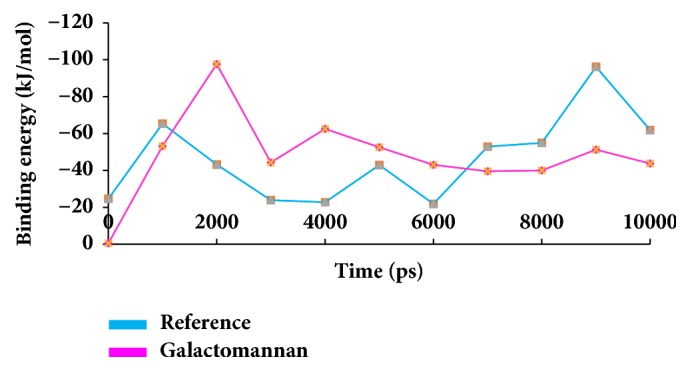
MM/PBSA binding energy representation of the reference and the candidate compound.

Binding free energies are computed after the MD simulations that inspect protein fluctuations and ligand conformations thereby ensuring a suitable positioning of the ligand within the binding site. The MM/PBSA calculations have produced a favourable ΔG that ranged between -10 to 100 kJ/mol for breast cancer target, as in Figure 15. Furthermore, the average binding energy produced by reference was -42.45 kJ/mol while that generated by galactomannan was -47.95 kJ/mol, respectively, as in Table 6.

**Figure 16:**
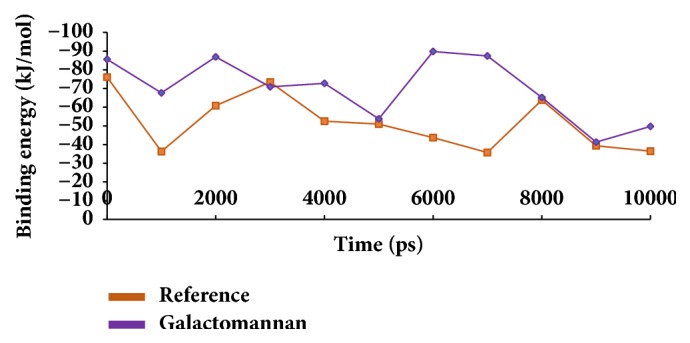
MM/PBSA binding energy representation of the reference and the candidate compound.

The binding free energies were subsequently calculated for canagliflozin-protein and galactomannan-protein systems for type 2 diabetes. 10 snapshots were evenly extracted and the binding energies were computed accordingly. The binding energies ranged between -15 kJ/mol and -100 kJ/mol, as in Figure 16. Additionally, it was observed that the average binding energy was calculated as -51.75 kJ/mol for the reference and -68.11 kJ/mol for galactomannan, as in Table 7.

From the results, it is evident that galactomannan has represented higher –CDOCKER interaction energy values and lower binding free energies than their respective reference compounds. These results demonstrate that galactomannan has stronger binding affinities than the reference inhibitors.

## 4. Discussion

In the present study, the seed extract showed the presence of proteins, carbohydrates, fatty acids, oils, saponin, flavonoids, tannins, terpenoids, alkaloids, soluble fibre galactomannan, and amino acid 4 hydroxyisoleusine (Tables 1 and 2). Some chemicals screened are similar to the work done by Yadav R. et al. 2014 [63].

Out of these chemicals, the special interest in this investigation is on the percentages of soluble fibre galactomannan 26.4 % and free amino acids 4 hydroxyleucine 13% and the presence saponins, as these are linked to human health benefits mainly in the reduction of plasma glucose levels and anticancer activities [64].

In order to further evaluate molecular inhibitory effect of the selected phytochemicals, the investigation proceeds* in silico*. Computational results have revealed that the phytochemical 4 hydroxyisoleucine could not induce the inhibitory activity against both the diseases. Although reports exist to explain its antidiabetic and antibreast cancer activity, the present finding foretells its inability as an inhibitor [31, 65]. Therefore, this amino acid was not forwarded for further studies. The other compound galactomannan has proved to be potential against both the diseases. This was represented by the RMSD, RMSF, and the potential energy values. The results were found to be unaltered as compared with the reference throughout the simulations. Moreover, the binding energies of the prospective drug molecules are found to be less, while rendering the highest –CDOCKER interaction energies. It is documented from the previous reports regarding the role of breast cancer inhibitors on diabetes mellitus as there exists a linkage between them [66, 67]. All the above results conclude that galactomannan could be considered as a potential drug for both the diseases.

Chemically, galactomannan is a polysaccharide molecule comprising a mannose backbone and the galactose side groups, hence, the name. More precisely they exist with 1-6 alpha-D-galactopyranose linkage. However, in fenugreek, mannose and galactose are linked by 1:1 linkage. Upon observing the docking conformations, it can be elucidated that the galactose side groups have involved in forming the hydrogen bond interaction with the active side residues, with the ring structures of the mannose involved in the formation of the *π* bond interactions.

In conclusion, the present study has examined the active components of fenugreek seeds against two common but different diseases,* viz-a-viz*: type-2 diabetes and breast cancer, using a well-established computational drug discovery method. The chemical composition of fenugreek seeds was assessed, and galactomannan and 4-hydroxyisoleucine were identified as major components and are similar to previous studies [68]. The therapeutic potential of these two identified active components was further assessed using molecular docking and molecular dynamics simulations. Our results identify galactomannan as a potential active component of fenugreek seeds, with a docking score compared to established drugs such as canagliflozin and anastrozole in binding simulations of therapeutics against type-2 diabetes and breast cancer, respectively. These results establish galactomannan, derived from fenugreek seeds, as a potential candidate for further drug discovery experiments in establishing their value as therapeutics against type-2 diabetes and breast cancer.

## Figures and Tables

**Table 1:** Percentage of the seed contents.

Contents of fenugreek seed extract	Average percentage of the seed extracts (%)
protein	28.5
carbohydrate	16.2
oils	5.3
fatty acid	3.8
galactomannan	26.4
moisture	6.8
4-hydroxyisoleucine	13

**Table 2:** Summary of Phytochemicals in Acetone Extract of Fenugreek Seed.

Tests	Results
Flavanoid	+ve
Tannin	+ve
Terpenoids	+ve
Alkaloids	+ve
Saponins	+ve

**Table 3:** Molecular dock scores between the drug targets and the compounds.

**S. no.**	**Name of the compound**	**-CDOCKER interaction energy**
Dock scores of diabetes mellitus		
**1**	canagliflozin	36.55
**2**	galactomannan	43.19
**3**	4-hydroxyisoleucine	28.27
Dock scores of breast cancer		
**1**	anastrozole	34.05
**2**	galactomannan	58.15
**3**	4-hydroxyisoleucine	23.88

**Table 4:** The molecular interactions between the compounds and the protein.

S. no.	Compound	Ligand Atom	Amino acid	Amino acid atom	Bond length (Å)	Hydrophobic interactions
1	anastrozole	N5	Met374	HN	2.9	Ile133,Asp309,Val370, Leu372, Val373,Pro429,Phe430,Cys437,Leu 477
2	galactomannan	O13	Arg115	HH22	2.8	Ala306, Asp309, Phe430.
		O15	Arg115	HH21	2.5	
		H62	Ile132	O	2.6	
		O14	Arg145	HH11	2.0	
		H57	Asp309	OD2	2.8	
		H53	Met374	O	2.5	
		H63	Cys437	SG	2.5	

**Table 5:** The molecular interactions between the compounds and the protein.

S.no	Compound	Ligand Atom	Amino acid	Amino acid atom	Bond length Å	Hydrophobic interactions
1	canagliflozin	F2	Cys215	HG	2.6	Lys120,Lys116, Ser216,Gly218, Ile219,Gly220, Ala217,Gln262
		F2	Arg221	HN	2.1	
		F2	Arg221	HE	2.1	
2	galactomannan	O9	Lys120	HZ2	1.7	Tyr46,Lys116, Phe182,Gly183, Cys215,Ser216, Gly218,Ile219, Gly220
		O3	Lys120	HZ1	2.0	
		H66	Asp181	OD1	2.3	
		H64	Asp181	OD1	1.9	
		O14	Arg221	HN	2.4	
		O16	Ser216	HN	2.4	
		H62	Gln262	OE1	2.1	
		O13	Gln266	HE22	2.4	

**Table 6:** Comparative assessment between dock scores and the binding energies of breast cancer systems.

S. no.	Name of the compound	-CDOCKER interaction energy	Average binding energy (kJ/mol)
1	anastrozole	34.05	-42.45
2	galactomannan	58.15	-47.95

**Table 7:** Comparative assessment between dock scores and the binding energies type 2 diabetes systems.

S. no.	Name of the compound	-CDOCKER interaction energy	Average binding energy (kJ/mol)
1	canagliflozin	36.55	-51.75
2	galactomannan	43.19	-68.11

## Data Availability

The data used to support the findings of this study are available from the corresponding author upon request

## References

[1] Nagulapalli Venkata K. C., Swaroop A., Bagchi D., Bishayee A. (2017). A small plant with big benefits: Fenugreek (Trigonella foenum-graecum Linn.) for disease prevention and health promotion. *Molecular Nutrition & Food Research*.

[2] Poole C., Bushey B., Foster C. (2010). The effects of a commercially available botanical supplement on strength, body composition, power output, and hormonal profiles in resistance-trained males. *Journal of the International Society of Sports Nutrition*.

[3] Shabbeer S., Sobolewski M., Anchoori R. K. (2009). Fenugreek: a naturally occurring edible spice as an anticancer agent. *Cancer Biology & Therapy*.

[4] Wani S. A., Kumar P. (2018). Fenugreek: A review on its nutraceutical properties and utilization in various food products. *Journal of the Saudi Society of Agricultural Sciences*.

[5] Basch E., Ulbricht C., Kuo G., Szapary P., Smith M. (2003). Therapeutic applications of fenugreek. *Alternative Medicine Review*.

[6] Tiran D. (2003). The use of fenugreek for breast feeding women. *Complementary Therapies in Nursing and Midwifery*.

[7] Szabó K., Gesztelyi R., Lampé N. (2018). Fenugreek (Trigonella Foenum-Graecum) Seed Flour and Diosgenin Preserve Endothelium-Dependent Arterial Relaxation in a Rat Model of Early-Stage Metabolic Syndrome. *International Journal of Molecular Sciences*.

[8] Sharma N., Suresh S., Debnath A., Jha S. (2017). Trigonella seed extract ameliorates inflammation via regulation of the inflammasome adaptor protein, ASC. *Frontiers in Bioscience - Elite*.

[9] Pundarikakshudu K., Shah D. H., Panchal A. H., Bhavsar G. C. (2016). Anti-inflammatory activity of fenugreek (*Trigonella foenum-graecum Linn*) seed petroleum ether extract. *Indian Journal of Pharmacology*.

[10] Sethi G., Shanmugam M., Warrier S. (2018). Pro-Apoptotic and Anti-Cancer Properties of Diosgenin: A Comprehensive and Critical Review. *Nutrients*.

[11] Shivashankara A. R., Azmidah A., Haniadka R., Rai M. P., Arora R., Baliga M. S. (2012). Dietary agents in the prevention of alcohol-induced hepatotoxicty: Preclinical observations. *Food & Function*.

[12] Kaviarasan S., Anuradha C. V. (2007). Fenugreek (*Trigonella foenum* graecum) seed polyphenols protect liver from alcohol toxicity: a role on hepatic detoxification system and apoptosis. *Die Pharmazie*.

[13] Bano D., Tabassum H., Ahmad A., Mabood A., Ahmad I. Z. (2016). The medicinal significance of the bioactive compounds of trigonella foenum-graecum: a review. *International Journal of Research in Ayurveda & Pharmacy*.

[14] Goyal S., Gupta N., Chatterjee S. (2016). Investigating therapeutic potential of trigonella foenum-graecum L. As our defense mechanism against several human diseases. *Journal of Toxicology*.

[15] Premanath R., Sudisha J., Devi N. L., Aradhya S. M. (2011). Antibacterial and anti-oxidant activities of fenugreek (Trigonella foenum graecum L.) leaves. *Research Journal of Medicinal Plant*.

[16] Haouala R., Hawala S., El-Ayeb A., Khanfir R., Boughanmi N. (2008). Aqueous and organic extracts of Trigonella foenum-graecum L. inhibit the mycelia growth of fungi. *Journal of Environmental Sciences*.

[17] Ahmad A., Alghamdi S. S., Mahmood K., Afzal M. (2016). Fenugreek a multipurpose crop: Potentialities and improvements. *Saudi Journal of Biological Sciences*.

[18] Altuntaş E., Özgöz E., Taşer Ö. F. (2005). Some physical properties of fenugreek (Trigonella foenum-graceum L.) seeds. *Journal of Food Engineering*.

[19] Rizvi S., Mishra N. (2013). Traditional Indian Medicines Used for the Management of Diabetes Mellitus. *Journal of Diabetes Research*.

[20] Kirkman M. S., Briscoe V. J., Clark N. (2012). Diabetes in older adults. *Diabetes Care*.

[21] Schneider H., Shaw J., Zimmet P. (2003). Guidelines for the Detection of Diabetes Mellitus - Diagnostic Criteria and Rationale for Screening. *The Clinical Biochemist Reviews*.

[22] Bellamy L., Casas J. P., Hingorani A. D., Williams D. (2009). Type 2 diabetes mellitus after gestational diabetes: a systematic review and meta-analysis. *The Lancet*.

[23] Moghadam F. H., Vakili-Zarch B., Shafiee M., Mirjalili A. (2013). Fenugreek seed extract treats peripheral neuropathy in pyridoxine induced neuropathic mice. *EXCLI Journal*.

[24] Gaddam A., Galla C., Thummisetti S., Marikanty R. K., Palanisamy U. D., Rao P. V. (2015). Role of Fenugreek in the prevention of type 2 diabetes mellitus in prediabetes. *Journal of Diabetes and Metabolic Disorders*.

[25] Madar Z., Abel R., Samish S., Arad J. (1988). Glucose-lowering effect of fenugreek in non-insulin dependent diabetics. *European Journal of Clinical Nutrition*.

[26] Attokaran M. *Effectiveness of phytotherapy in supportive treatment of type 2 diabetes mellitus II. Fenugreek (Trigonella foenum-graecum)*.

[27] Sharma R. D., Raghuram T. C., Rao N. S. (1990). Effect of fenugreek seeds on blood glucose and serum lipids in type I diabetes. *European Journal of Clinical Nutrition*.

[28] Kumar G. S., Shetty A. K., Sambaiah K., Salimath P. V. (2005). Antidiabetic property of fenugreek seed mucilage and spent turmeric in streptozotocin-induced diabetic rats. *Nutrition Research*.

[29] Neelakantan N., Narayanan M., De Souza R. J., Van Dam R. M. (2014). Effect of fenugreek (Trigonella foenum-graecum L.) intake on glycemia: A meta-analysis of clinical trials. *Nutrition Journal *.

[30] Khoja K. K., Shafi G., Hasan T. N. (2011). Fenugreek, a naturally occurring edible spice, kills MCF-7 human breast cancer cells via an apoptotic pathway. *Asian Pacific Journal of Cancer Prevention*.

[31] Khalil M. I. M., Ibrahim M. M., El-Gaaly G. A., Sultan A. S. (2015). *Trigonella foenum* (Fenugreek) Induced Apoptosis in Hepatocellular Carcinoma Cell Line, HepG2, Mediated by Upregulation of p53 and Proliferating Cell Nuclear Antigen. *BioMed Research International*.

[32] Vígh S., Zsvér-Vadas Z., Pribac C. (2016). Fenugreek (Trigonella foenum-graecum l.) extracts are inducing dose-dependent hormetic response and cytotoxic effects in case of human breast cancer cell lines. *Studia Universitatis Vasile Goldis Arad, Seria Stiintele Vietii*.

[33] Rampogu S., Son M., Baek A. (2018). Targeting natural compounds against {HER}2 kinase domain as potential anticancer drugs applying pharmacophore based molecular modelling approaches. *Computational Biology and Chemistry*.

[34] Ferlay J., Shin H. R., Bray F., Forman D., Mathers C., Parkin D. M. (2010). Estimates of worldwide burden of cancer in 2008: GLOBOCAN 2008. *International Journal of Cancer*.

[35] Petrucelli N., Daly M. B., Feldman G. L. (2010). Hereditary breast and ovarian cancer due to mutations in BRCA1 and BRCA2. *Genetics in Medicine*.

[36] Amin A., Alkaabi A., Al-Falasi S., Daoud S. A. (2005). Chemopreventive activities of *Trigonella foenum graecum* (Fenugreek) against breast cancer. *Cell Biology International*.

[37] Wang G., Zhu W. (2016). Molecular docking for drug discovery and development: a widely used approach but far from perfect. *Future Medicinal Chemistry*.

[38] Rampogu S., Baek A., Zeb A., Lee K. W. (2018). Exploration for novel inhibitors showing back-to-front approach against VEGFR-2 kinase domain (4AG8) employing molecular docking mechanism and molecular dynamics simulations. *BMC Cancer*.

[39] Meng X.-Y., Zhang H.-X., Mezei M., Cui M. (2011). Molecular docking: a powerful approach for structure-based drug discovery. *Current Computer-Aided Drug Design*.

[40] Wang H., Pampati N., McCormick W. M., Bhattacharyya L. (2016). Protein nitrogen determination by kjeldahl digestion and ion chromatography. *Journal of Pharmaceutical Sciences*.

[41] Loewus F. A. (1952). Improvement in Anthrone Method for Determination of Carbohydrates. *Analytical Chemistry*.

[42] Paech K., Tracey M. V. (1955). *Modern Methods of Plant Analysis / Moderne Methoden der Pflanzenanalyse*.

[43] Rolland-Fulcrand V., Rolland M., Roumestant M.-L., Martinez J. (2004). Chemoenzymatic synthesis of enantiomerically pure (2S,3R,4S)-4- hydroxyisoleucine, an insulinotropic amino acid isolated from fenugreek seeds. *European Journal of Organic Chemistry*.

[44] Kooiman P. (1971). Structures of the galactomannans from seeds of Annona muricata, Arenga saccharifera, Cocos nucifera, Convolvulus tricolor, and Sophora japonica. *Carbohydrate Research*.

[45] Nielsen S. S. (2010). Determination of Moisture Content. *Food Analysis Laboratory Manual*.

[46] Ghosh D., Griswold J., Erman M., Pangborn W. (2009). Structural basis for androgen specificity and oestrogen synthesis in human aromatase. *Nature*.

[47] Peters G. H., Iversen L. F., Branner S. (2000). Residue 259 Is a Key Determinant of Substrate Specificity of Protein-tyrosine Phosphatases 1B and *α*. *The Journal of Biological Chemistry*.

[48] Toderika Y., Ferguson N. (2014). Canagliflozin: A new class of antidiabetic agent targeting the sodium-glucose cotransporter. *Cardiology in Review*.

[49] Sanford M., Plosker G. L. (2008). Anastrozole: A review of its use in postmenopausal women with early-stage breast cancer. *Drugs*.

[50] Rampogu S., Rampogu Lemuel M. (2016). Network Based Approach in the Establishment of the Relationship between Type 2 Diabetes Mellitus and Its Complications at the Molecular Level Coupled with Molecular Docking Mechanism. *BioMed Research International*.

[51] Zoete V., Cuendet M. A., Grosdidier A., Michielin O. (2011). SwissParam: a fast force field generation tool for small organic molecules. *Journal of Computational Chemistry*.

[52] Rampogu S., Son M., Park C., Kim H., Suh J., Lee K. (2017). Sulfonanilide Derivatives in Identifying Novel Aromatase Inhibitors by Applying Docking, Virtual Screening, and MD Simulations Studies. *BioMed Research International*.

[53] Rampogu S., Baek A., Son M. (2017). Computational Exploration for Lead Compounds That Can Reverse the Nuclear Morphology in Progeria. *BioMed Research International*.

[54] van der Spoel D., Lindahl E., Hess B., Groenhof G., Mark A. E., Berendsen H. J. C. (2005). GROMACS: fast, flexible, and free. *Journal of Computational Chemistry*.

[55] Hess B., Bekker H., Berendsen H. J. C., Fraaije J. G. E. M. (1997). LINCS: a linear Constraint Solver for molecular simulations. *Journal of Computational Chemistry*.

[56] Darden T., York D., Pedersen L. (1993). Particle mesh Ewald: an N·log(N) method for Ewald sums in large systems. *The Journal of Chemical Physics*.

[57] Berendsen H. J. C., Postma J. P. M., Van Gunsteren W. F., Dinola A., Haak J. R. (1984). Molecular dynamics with coupling to an external bath. *The Journal of Chemical Physics*.

[58] Parrinello M., Rahman A. (1981). Polymorphic transitions in single crystals: a new molecular dynamics method. *Journal of Applied Physics*.

[59] Humphrey W., Dalke A., Schulten K. (1996). VMD: visual molecular dynamics. *Journal of Molecular Graphics*.

[60] Baker N. A., Sept D., Joseph S., Holst M. J., McCammon J. A. (2001). Electrostatics of nanosystems: application to microtubules and the ribosome. *Proceedings of the National Acadamy of Sciences of the United States of America*.

[61] Kumari R., Kumar R., Lynn A. (2014). *g_mmpbsa*—A GROMACS tool for high-throughput MM-PBSA calculations. *Journal of Chemical Information and Modeling*.

[62] Hossain M. A., AL-Raqmi K. A. S., AL-Mijizy Z. H., Weli A. M., Al-Riyami Q. (2013). Study of total phenol, flavonoids contents and phytochemical screening of various leaves crude extracts of locally grown Thymus vulgaris. *Asian Pacific Journal of Tropical Biomedicine*.

[63] Yadav R., Tiwari R., Chowdhary P., Pradhan C. K. (2011). A pharmacognostical monogroaph of Trigonella foenum-graecum seeds. *International Journal of Pharmacy and Pharmaceutical Sciences*.

[64] Madar Z., Shomer I. (1990). Polysaccharide Composition of a Gel Fraction Derived from Fenugreek and Its Effect on Starch Digestion and Bile Acid Absorption in Rats. *Journal of Agricultural and Food Chemistry*.

[65] Zafar M. I., Gao F. (2016). 4-Hydroxyisoleucine: A Potential New Treatment for Type 2 Diabetes Mellitus. *BioDrugs*.

[66] Rampogu S. D. V. (2015). Role of breast cancer inhibitors on diabetes mellitus- an in silico approach. *Journal of Diabetes and Metabolic Disorders*.

[67] Ahmadieh H., Azar S. T. (2013). Type 2 Diabetes Mellitus, Oral Diabetic Medications, Insulin Therapy, and Overall Breast Cancer Risk. *ISRN Endocrinology*.

[68] Thomas J. E., Bandara M., Lee E. L., Driedger D., Acharya S. (2011). Biochemical monitoring in fenugreek to develop functional food and medicinal plant variants. *New Biotechnology*.

